# Racial disparities in COVID-19 pandemic cases, hospitalisations, and deaths: A systematic review and meta-analysis

**DOI:** 10.7189/jogh.11.05015

**Published:** 2021-06-26

**Authors:** William Mude, Victor M Oguoma, Tafadzwa Nyanhanda, Lillian Mwanri, Carolyne Njue

**Affiliations:** 1 School of Health, Medical and Applied Sciences, Central Queensland University, Cairns, Australia; 2 Health Research Institute, University of Canberra, Canberra, Australia; 3 School of Health, Medical and Applied Sciences, Central Queensland University, Melbourne, Australia; 4 College of Medicine and Public Health, Flinders University, Adelaide, Australia; 5 The Australian Centre for Public and Population Health Research (ACPPHR), University of Technology Sydney, Sydney, Australia

## Abstract

**Background:**

People from racial minority groups in western countries experience disproportionate socioeconomic and structural determinants of health disadvantages. These disadvantages have led to inequalities and inequities in health care access and poorer health outcomes. We report disproportionate disparities in prevalence, hospitalisation, and deaths from COVID-19 by racial minority populations.

**Methods:**

We conducted a systematic literature search of relevant databases to identify studies reporting on prevalence, hospitalisations, and deaths from COVID-19 by race groups between 01 January 2020 – 15 April 2021. We grouped race categories into Blacks, Hispanics, Whites and Others. Random effects model using the method of DerSimonian and Laird were fitted, and forest plot with respective ratio estimates and 95% confidence interval (CI) for each race category, and subgroup meta-regression analyses and the overall pooled ratio estimates for prevalence, hospitalisation and mortality rate were presented.

**Results:**

Blacks experienced significantly higher burden of COVID-19: prevalence ratio 1.79 (95% confidence interval (CI) = 1.59-1.99), hospitalisation ratio 1.87 (95% CI = 1.69-2.04), mortality ratio 1.68 (95% CI = 1.52-1.83), compared to Whites: prevalence ratio 0.70 (95% CI = 0.0.64-0.77), hospitalisation ratio 0.74 (95% CI = 0.65-0.82), mortality ratio 0.82 (95% CI = 0.78-0.87). Also, Hispanics experienced a higher burden: prevalence ratio 1.78 (95% CI = 1.63-1.94), hospitalisation ratio 1.32 (95% CI = 1.08-1.55), mortality ratio 0.94 (95% CI = 0.84-1.04) compared to Whites. A higher burden was also observed for Other race groups: prevalence ratio 1.43 (95% CI = 1.19-1.67), hospitalisation ratio 1.12 (95% CI = 0.89-1.35), mortality ratio 1.06 (95% CI = 0.89-1.23) compared to Whites. The disproportionate burden among Blacks and Hispanics remained following correction for publication bias.

**Conclusions:**

Blacks and Hispanics have been disproportionately affected by COVID-19. This is deeply concerning and highlights the systemically entrenched disadvantages (social, economic, and political) experienced by racial minorities in western countries; and this study underscores the need to address inequities in these communities to improve overall health outcomes.

In December 2019, a new pneumonia-like infection with varying symptoms, ranging from mild to severe shortness of breath, emerged from Wuhan, China [1]. A World Health Organisation (WHO) investigation designated the infection as a 2019 novel coronavirus and was subsequently named COVID-19 [2]. The infection quickly spread throughout the world; at the time of writing, the source has not yet been determined. It was declared a public health emergency of international concern by WHO in January 2020 and became a pandemic in March [3,4]. Transmission occurs through air droplets, and no proven cure had existed against the virus. As of 24 May 2021 at 2:50 pm Central European Summer Time (CEST), there have been 166 860 081 confirmed COVID-19 infection cases and 3 459 996 related deaths worldwide and rising [5].

Available evidence suggests that medical comorbidities, obesity, diabetes, old age, and being a male are risk factors for COVID-19 [6,7]. However, in countries where data has been reported for race, the data shows that the burdens of COVID-19 are disproportionately high among racial minority groups [6,8]. For example, in the United Kingdom, the United States of America, and Brazil, high cases of COVID-19 are reported in people from racial minority groups [9-12]. The reports show that in the United Kingdom and the United States of America, 35% and 33% of COVID-19 patients, respectively, are from racial minority populations [7,10], although these populations make much lower proportions of the total population. Some experts have claimed these disproportionately high burdens of COVID-19 are a result of health disparities and entrenched inequities experienced by minority communities [13]. However, conflicting findings relating to the reported burden of COVID-19 by racial groups have shown different rates. Some reports have claimed that White populations have higher mortality than Blacks, Asian, and Minority Ethnic (BAME) groups, while others showed that BAME groups have higher cases or no differences [14-16]. A recently published systematic review by Pan and colleagues [17] suggests that Blacks have a high risk of acquiring COVID-19 infection and worse clinical outcomes than Whites. However, the systematic review conducted by Pan and colleagues [17] did not run a meta-analysis and only summarised finding from each of the studies without synthesising them to highlight the extent of the issue.

In this article, we report our systematic review and meta-analysis of the literature on the burden of COVID-19 by race. We assessed the disparity of COVID-19 prevalence, hospitalisation and mortality ratios among Blacks, Hispanics, Whites, and Other race groups. This review provides vital information on the burdens of COVID-19 among the selected race group and will help support policies that address health inequities.

## METHODS

### Search strategy

Our search strategy was guided by the PRISMA statement [18]. We conducted the systematic search strategy in the following databases and conference proceedings: CINAHL Complete, Medline, Web of Science (Science Citation Index Expanded (SCI-EXPANDED), Social Sciences Citation Index (SSCI), Arts & Humanities Citation Index (A&HCI), Conference Proceedings Citation Index- Science (CPCI-S), Conference Proceedings Citation Index- Social Science & Humanities (CPCI-SSH), and Emerging Sources Citation Index (ESCI)) for peer-reviewed papers published between 1 January 2020 – 15 April 2021. We searched these databases and conference proceedings using the search terms: TI(prevalence OR incidence OR burden* OR rate* OR death* OR Mortali*) AND (COVID-19 OR 2019 novel coronavirus disease OR COVID19 OR COVID-19 pandemic OR SARS-CoV-2 infection OR COVID-19 virus disease OR 2019 novel coronavirus infection OR 2019-nCoV infection OR coronavirus disease 2019 OR coronavirus disease-19 OR 2019-nCoV disease OR COVID-19 virus infection) AND (people of colo* OR minor* OR immigrant* OR African American OR Hispanics OR black* OR emigrant* OR ethnic). We also searched grey literature and government websites for COVID-19 data by race.

### Outcomes

The primary outcomes assessed were COVID-19 prevalence, hospitalisations, and deaths by the following selected race categories: Blacks, Hispanics, Whites, and Other race groups. Blacks were defined as people with African ancestral origins who self-identify or are identified as Black, African or Afro-Caribbean [19,20]. Whites were described as people with European ancestral origins who identify or are identified as White non-Hispanic [20]. Hispanics were defined as people of Spanish speaking backgrounds from Central and South America who identify or are identified by others as Hispanic or Latino. Other race groups were defined as race groups not identified as Whites, Hispanics or Blacks (Asians, Indigenous, mixed-race, and unknown). Few COVID-19 studies have categorised Asians as a separate racial group when examining COVID-19 outcomes by race. Because of this, we have grouped Asian in “Other race groups”.

### Screening and appraisal

**Figure 1:**
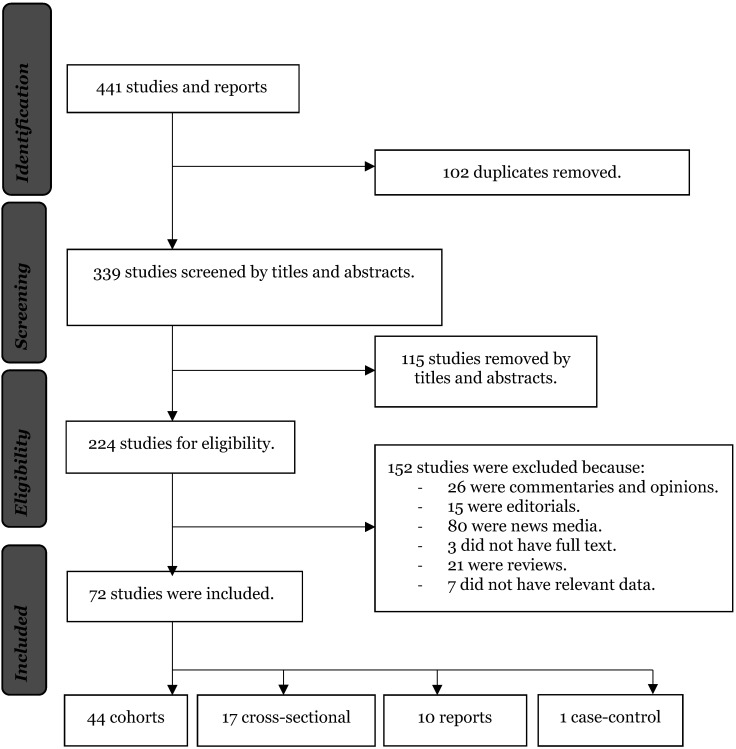
Flow diagram of studies included in the review.

The lead author, the third co-author and the last co-author conducted the search and screening process separately, identifying studies by titles and abstracts. Studies that reported race data on prevalence or deaths or hospitalisations were included in this study. Studies were excluded if they were commentaries, letters, reviews, and opinions. At the same time, studies that reported prevalence, deaths, and hospitalisation for other health conditions were excluded. After removing the duplicates, studies were screened by titles, abstracts and read in full. Studies that met the inclusion criteria (**Figure 1**) were appraised for quality using the JBI quality appraisal for systematic literature reviews [21].

### Data extraction and meta-analysis

We independently extracted information from studies that met the inclusion criteria. Information on the author, year of publication, country, study design, race, population proportion, samples, cases, prevalence, deaths, and hospitalisation was extracted, where appropriate. We separated the studies into four different race groups: Blacks, Hispanics, Whites, and Other race groups (Asians, Indigenous, mixed-race, and others).

We calculated the expected number of cases, hospitalisations, and deaths for each study, where appropriate. We used the expected number of cases, hospitalisations, and deaths to calculate the prevalence, hospitalisation, and mortality rate ratios weighted by the population of each race group. Where population proportion for race category was not provided, we used respective country census data (USA [22] and UK [23]) to determine each race proportion to establish weighted prevalence ratio, hospitalisation ratio, and mortality rate.

We assumed that the probability for the occurrence of the expected cases, hospitalisations and deaths was the same as the probabilities for the occurrence of the observed cases, hospitalisations, and deaths in the absence of any changes in the rate of infectivity and the risk of exposure. We also assumed that all members of the population were at risk of infection, hospitalisations, and deaths from COVID-19 because no vaccination had existed at the time and that the pattern of the outbreak was random. That is, people randomly took up testing for COVID-19 from drive-by testing centres and hospitals, and therefore the reported samples were representative of the population group.

We calculated 95% confidence intervals (CI) using both the exact Poisson distribution approach for observed and expected counts <100 and the approach for observed and expected counts >100 [24]. Random effects model using DerSimonian and Laird’s methods were fitted, and forest plot with respective ratio estimates and 95% CI were presented for each race category in subgroup analyses and the overall pooled ratio estimates for prevalence, hospitalisation and mortality. The -statistic was used to test the overall effect with statistical significance set at α≤0.005 Heterogeneity between studies was assessed using the *I^2^* statistic. Nonparametric trim-and-fill analysis was used to assess for publication bias [25,26]. Multivariable meta-regression was performed to explore heterogeneity resulting from the relationship between study effect sizes and race, country, year, and study design. The metan Stata module in Stata 16.1 MP (StataCorp, College Station, TX, USA) was used to conduct the meta-analysis [27].

## RESULTS

### Studies

We included 72 studies that reported at least observed cases, hospitalisations, or deaths from COVID-19. The included studies and the period covered by each study are provided in Table S1 in the **Online Supplementary Document**. Fifty-four studies (75.0%) were from the United States of America, 13 (18.1%) from the United Kingdom, and five (6.9%) from Brazil. Forty-four studies were cohorts (61.1%), 10 reports (13.9%), 17 cross-sectional (23.6%) and one case-control (1.4%). Twelve studies (16.2%) reported the observed cases, hospitalisations, and deaths [28-40]. Ten studies (13.5%) reported observed hospitalisations and deaths [41-50], and eight studies (12.2%) reported cases and deaths [34,51-58]. Two studies (2.8%) reported observed cases and hospitalisations [59,60]. Seventeen studies (22.6%) reported observed cases only [61-77]. Twelve studies (16.2%) reported hospitalisations [78-88], and 11 (14.9%) reported deaths only [12,89-98].

### COVID-19 prevalence ratio

**Figure 2:**
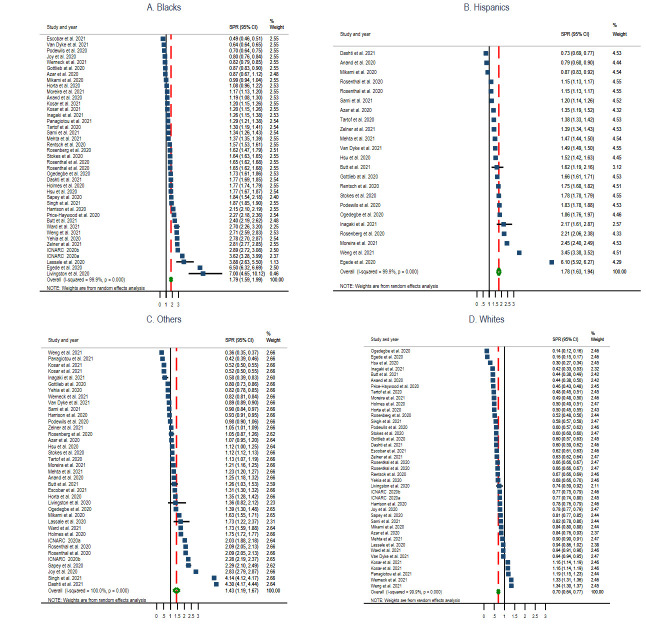
Standardised prevalence ratio (SPR) Forest Plots of COVID-19 by selected race groups. Panel A: Blacks. Panel B: Hispanics. Panel C: Other race groups. Panel D: Whites.

Thirty-nine studies (54.2%) (22 cohorts [28-32,34,36,40,52-57,59,62,63,66,67,73-75], ten cross-sectional [35,51,58,61,64,65,68-70,72], six reports [33,38,39,71,76,77], and one case-control study [60]) had data to calculate the standardised prevalence ratio of COVID-19 for the identified race categories in the general community. The pooled prevalence ratio for Blacks was 1.79 (95% CI = 1.59, 1.99; *I^2^* = 99.9%, *P* < 0.001) (**Figure 2**, Panel A), Hispanics 1.78 (95% CI = 1.63, 1.94; *I^2^* = 99.9%, *P* < 0.001) (**Figure 2****,** Panel B), Other race groups 1.43 (95% CI = 1.19, 1.67; *I^2^* = 100.0%, *P* < 0.001) (**Figure 2**, Panel C), and Whites 0.70 (95% CI = 0.64, 0.77; *I^2^* = 99.9%, *P* < 0.001) (**Figure 2**, Panel D).

**Figure 3:**
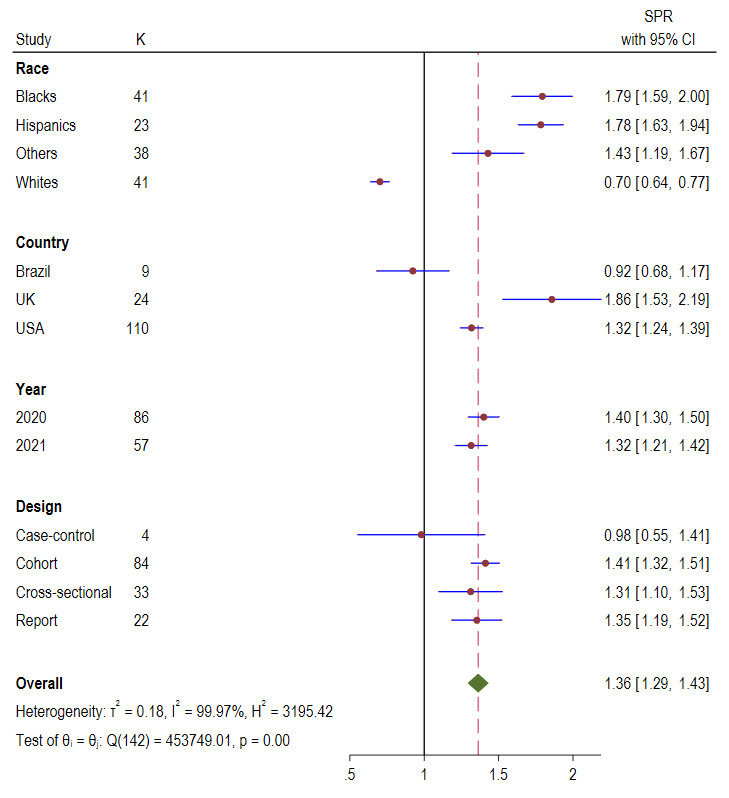
Standardised prevalence ratio (SPR) Forest plots of COVID-19 by race, country, year, and study design.

As shown in **Figure 3**, the overall pooled COVID-19 prevalence ratio for all population was 1.36 (95% CI = 1.29-1.43; *I^2^* = 99.97%, *P* < 0.001). Subgroup analysis showed that UK had the highest of COVID-19 prevalence ratio, 1.86 (95% CI = 1.53, 2.19), followed by USA, 1.32 (95% CI = 1.53, 2.19) and Brazil, 0.92 (95% CI = 0.68, 1.17). The COVID-19 prevalence ratio reported in studies published between the year 2020 and 2021 were 1.40 (95% CI = 1.30, 1.50) and 1.32 (95% CI = 1.21, 1.42), respectively. Cohort studies reported the highest COVID-19 prevalence ratio of 1.41 (95% CI = 1.32, 1.51) followed by reports 1.35 (95% CI = 1.19, 1.52) and cross-sectional studies 1.31 (95% CI = 1.10, 1.53).

### COVID-19 hospitalisation ratio

**Figure 4:**
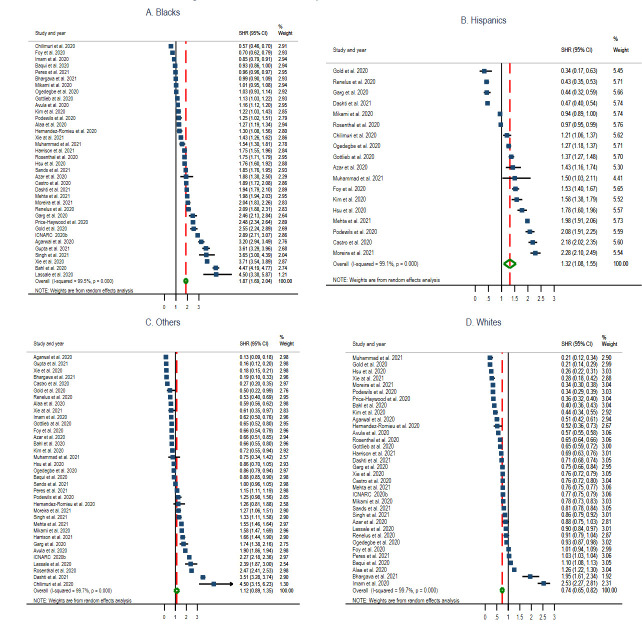
Standardised hospitalisation ratio (SHR) Forest plots of COVID-19 by selected race groups. Panel A: Blacks. Panel B: Hispanics. Panel C: Other race groups. Panel D: Whites.

Thirty-six studies (48.6%) (six reports [7,33,38,39,88], twenty-six cohorts [16,28-32,36,40,41,43-50,59,69,78-81,84,87,88], three cross-sectional [35,42,86], and one case-control [60]) reported data with information on hospitalisation by race. The pooled estimate of hospitalisation ratio among Blacks was 1.87 (95% CI = 1.69, 2.04; *I^2^* = 99.5%, *P* < 0.001) (**Figure 4**, Panel A), Hispanics 1.32 (95% CI = 1.08, 1.55; *I^2^* = 99.1%, *P* < 0.001) (**Figure 4**, Panel B), Other race groups 1.12 (95% CI = 0.89, 1.35; *I^2^* = 99.7%, *P* < 0.00) (**Figure 4**, Panel C), and Whites 0.74 (95% CI = 0.65, 0.82; *I^2^* = 99.7%, *P* < 0.001) (**Figure 4**, Panel D).

**Figure 5:**
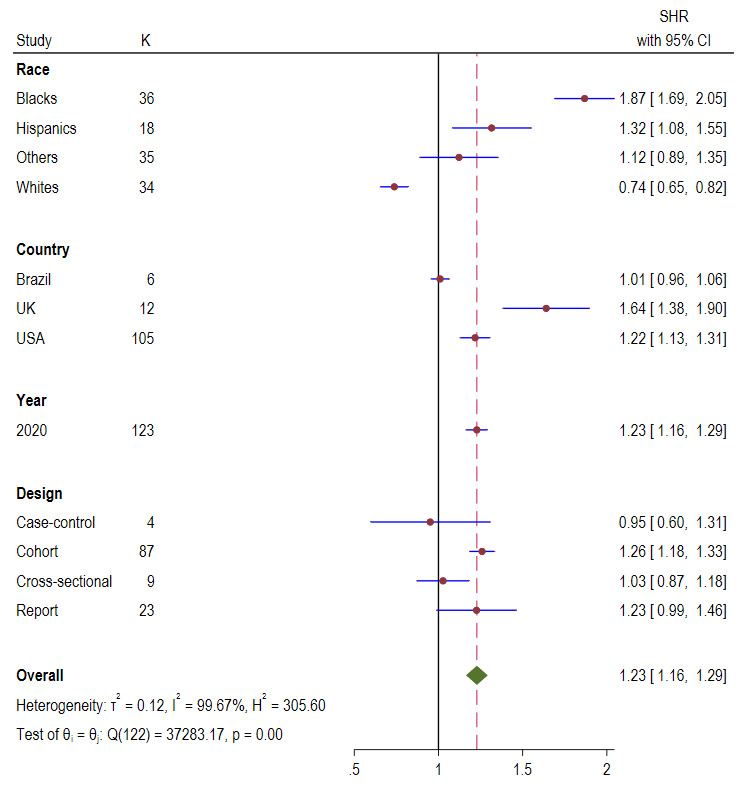
Standardised hospitalisation ratio (SHR) Forest plots of COVID-19 by race, country, year, and study design.

The pooled overall hospitalisation ratio was 1.23 (95% CI = 1.16, 1.29; *I^2^* = 99.67%, *P* < 0.001) (**Figure 5**). Country level analysis showed that UK experienced the highest hospitalisation ratio 1.64 (95% CI = 1.38, 1.90) followed by USA 1.22 (95% CI = 1.13, 1.31) and Brazil 1.01 (95% CI = 0.96, 1.06). Analysis by study designs found that cohort studies reported the highest hospitalisation ratio, 1.26 (95% CI = 1.18, 1.33), followed by cross-sectional 1.03 (95% CI = 0.87, 1.18), reports 1.23 (95% CI = 0.99-1.46) and case-control 0.95 (95% CI = 0.60, 1.31).

### COVID-19 mortality ratio

**Figure 6:**
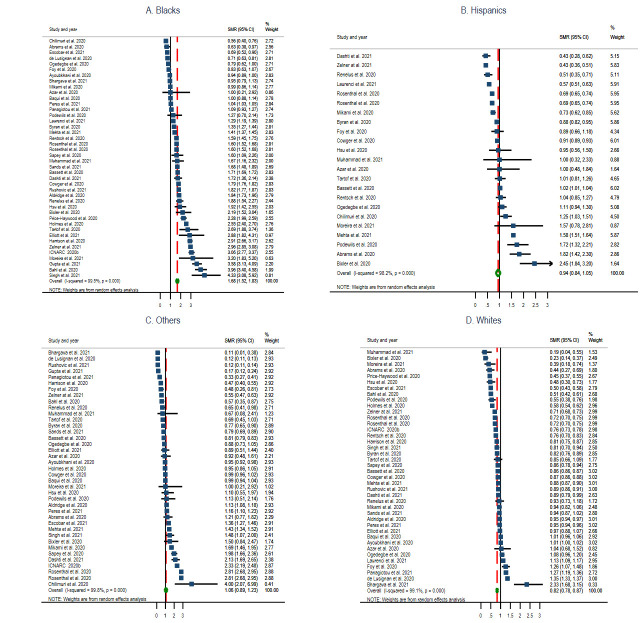
Standardised mortality ratio (SMR) Forest plots of COVID-19 by selected race groups. Panel A: Blacks. Panel B: Hispanics. Panel C: Other race groups. Panel D: Whites.

Forty-two studies (56.8%) (twenty-nine cohorts [12,28-32,34,36,40,41,43-50,52-57,89,90,93,96], four reports [38,39,41,90] and nine cross-sectional studies [35,42,51,58,91,94,95,97,98]) reported data with information to determine mortality rates by race groups. **Figure 6** presents the forest plots for mortality ratio by race. The plots showed that the pooled estimate for the mortality ratio in Blacks was 1.68 (95% CI = 1.52, 1.83; *I^2^* = 99.5%, *P* < 0.00) (**Figure 6**, Panel A), Hispanics 0.94 (95% CI = 0.84, 1.05; *I^2^* = 98.2%, *P* < 0.00) (**Figure 6**, Panel B), Other race groups 1.06 (95% CI = 0.89, 1.23; *I^2^* = 99.8%, *P* < 0.00) (**Figure 6**, Panel C), and Whites 0.82 (95% CI = 0.78, 0.87; *I^2^* = 99.1%, *P* < 0.00) (**Figure 6**, Panel D).

**Figure 7:**
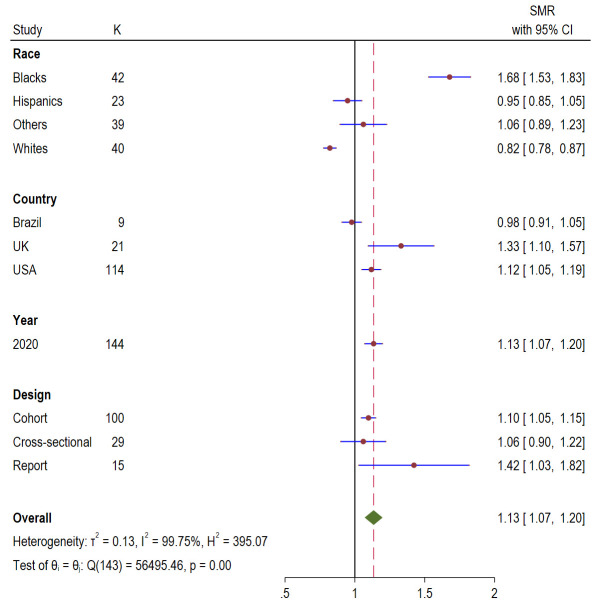
Standardised mortality ratio (SMR) Forest plots of COVID-19 by race, country, year, and study design.

The overall pooled mortality ratio for all population shown in **Figure 7** was 1.13 (95% CI = 1.07, 1.1.20; *I^2^* = 99.75%, *P* < 0.00). Mortality ratio by country was highest for UK 1.33 (95% CI = 1.10, 1.57), followed by USA 1.12 (95% CI = 1.05, 1.19), and Brazil 0.98 (95% CI = 0.98, 1.05). Report studies had the highest mortality ratio 1.42 (95% CI = 1.03, 1.82), followed by cohort 1.10 (95% CI = 1.05, 1.15), and cross-sectional 1.06 (95% CI = 0.90, 1.22).

### Meta-analysis and regression

**Table 1** shows the mean difference in COVID-19 outcomes for prevalence, hospitalisation, and mortality ratio. It was found that the prevalence ratio between Blacks and Whites was significant, -1.09 (95% CI = -1.28, -0.90; *P* < 0.000). A significant finding was also observed between Blacks and Other race groups, -0.35 (95% CI = -0.55, -0.16; *P* < 0.000). However, the difference in COVID-19 prevalence ratio between Blacks and Hispanics was not significant, 0.11 (95% CI = -0.12, 0.34; *P* = 0.34). For the mean difference in prevalence ratio by study designs, a significant difference was observed only between case-control and cohort study design, 0.48 (95% CI = 0.04, 0.93; *P* = 0.03). No significant mean difference is prevalence ratio was found between the studies published in 2020 and 2021, -0.07 (95% CI = -0.22, 0.09; *P* = 0.39).

**Table 1:** Meta-regression of Mean difference for prevalence, hospitalisation, and mortality ratios by subgroups

Outcomes	Parameters	Mean difference (95% CI)	*P* value
**Prevalence ratio**			
Race	Black	Ref	.
	Hispanics	0.11 (-0.12, 0.34)	0.343
	Other	-0.35 (-0.55, -0.16)	<0.001
	White	-1.09 (-1.28, -0.90)	<0.001
Country	Brazil	Ref	.
	UK	0.91 (0.56, 1.26)	<0.001
	USA	0.19 (-0.13, 0.51)	0.248
Year	2020	Ref	.
	2021	-0.07 (-0.22, 0.09)	0.387
Design	Case-control	Ref	.
	Cohort	0.48 (0.04, 0.93)	0.033
	Cross-sectional	0.25 (-0.22, 0.72)	0.302
	Report	0.24 (-0.23, 0.71)	0.312
**Hospitalisation ratio**			
Race	Black	Ref	.
	Hispanics	-0.49 (-0.73, -0.24)	<0.001
	Other	-0.77 (-0.97, -0.56)	<0.001
	White	-1.11 (-1.31, -0.91)	<0.001
Country	Brazil	Ref	.
	UK	0.67 (0.24, 1.09)	<0.001
	USA	0.11 (-0.26, 0.49)	0.558
Design	Case-control	0.00 (0.00, 0.00)	.
	Cohort	0.30 (-0.12, 0.72)	0.158
	Cross-sectional	0.11 (-0.42, 0.63)	0.691
	Report	0.21 (-0.24, 0.65)	0.366
**Mortality ratio**			
Race	Black	Ref	.
	Hispanics	-0.65 (-0.83, -0.47)	<0.001
	Other	-0.60 (-0.75, -0.44)	<0.001
	White	-0.82 (-0.97, -0.68)	0.00
Country	Brazil	Ref	.
	UK	0.27 (0.01, 0.53)	0.039
	USA	0.13 (-0.10, 0.35)	0.276
Design	Cohort	Ref	.
	Cross-sectional	-0.07 (-0.21, 0.07)	0.318
	Report	0.26 (0.05, 0.46)	0.014

CI – confidence interval

For hospitalisation ratio, it was found that the mean difference in hospitalisation ratio in Blacks was significantly different from Whites -1.11 (95% CI = -1.31, -0.91; *P* < 0.001), Hispanics -0.49 (95% CI = -0.73, -0.24; *P* < 0.001), and Other race groups -0.77 (95% CI = -0.97, -0.56; *P* < 0.001). Intercountry analysis showed COVID-19 mean difference in hospitalisation ratio was significant between Brazil and UK 0.67 (0.24, 1.09; *P* < 0.001) but not between Brazil and USA 0.11 (95% CI = -0.26, 0.49; *P* = 0.560). The mean difference in hospitalisation ratios between study designs were not significant.

The mean difference in mortality ratio between Blacks and Hispanics -0.65 (95% CI = -0.83, -0.47; *P* < 0.00), Blacks and Whites -0.82 (95% CI = -0.97, -0.68; *P* < 0.001), Blacks and Other race groups -0.60 (95% CI = -0.75, -0.44; *P* < 0.00) were all significant. Country level analysis showed that the mean difference between Brazil and UK was significant 0.27 (95% CI = 0.01, 0.53; *P* = 0.04), but no difference existed between Brazil and USA 0.13 (95% CI = -0.10, 0.35; *P* = 0.28). Analysis for study design found that the mean difference in mortality ratio between cohorts and reports was significant, 0.26 (95% CI = 0.05, 0.46; *P* = 0.01) but not between cohorts and cross-sectional studies, -0.07 (95% CI = -0.21, 0.07; *P* = 0.32).

### Publication bias

Following correction for publication bias (Appendix S1-S3 and Figures S1-S3 in the **Online Supplementary Document**), the prevalence ratio among Blacks and Hispanics remained high; 1.38 (95% CI = 1.19, 1.57) for Blacks and 1.59 (95% CI = 1.43, 1.75) for Hispanics compared to 0.69 (0.62, 0.75) for Whites and 1.01 (95% CI = 0.83, 1.37) for Other race groups. For hospitalisation ratio, Blacks and Hispanics continued to have a high hospitalisation ratio even when corrected for publication bias compared to Whites and Other race groups. For example, the hospitalisation ratio for Black was 1.42 (95% CI = 1.25, 1.59), Hispanics 1.32 (1.08-1.55) compared to 0.67 (95% CI = 0.59, 0.75) for Whites and 0.74 (0.49, 0.98) for Other race groups. The mortality ratio for Blacks remained high, 1.32 (1.17, 1.45), compared to Hispanics 0.82 (95% CI = 0.70, 0.93), Whites 0.82 (95% CI = 0.78, 0.87) and Other race groups 0.83 (95% CI = 0.67, 0.99) following correction for publication bias.

## DISCUSSION

The reviewed studies showed that COVID-19 significantly impacted Blacks across all the outcomes measured compared to Whites. The study found that the prevalence ratios in Blacks were 156% higher than in Whites, for Hispanics were 154% higher, and for Other race groups were 104% higher. There was a significant difference between prevalence ratios in Blacks and Whites and Other race groups but not Hispanics. Hospitalisation ratios in Blacks were 153% higher than in Whites, for Hispanics were 78% higher, and for Other race groups were 51% higher. A significant difference was found between hospitalisation in Blacks and Hispanics, Whites and Other race groups. Deaths in Blacks were 105% higher than in Whites, Hispanics were 15% higher, and Other race groups were 29% higher. Mortality in Blacks was significantly different from Whites, Hispanics, and Other race groups. Intercountry differences were also observed regarding prevalence ratios of COVID-19. The prevalence ratio in the USA was 102% higher than Brazil’s, and UK’s was 43% higher than Brazil’s. Although Blacks and Hispanics experienced a similar burden of COVID-19, Blacks had higher hospitalisation and mortality ratios than Hispanics, Whites and Other race groups.

The identified racial disparities in prevalence, hospitalisations and mortality ratio from COVID-19 could be attributed to several reasons. It could be that Blacks, Hispanics, and Other racial groups experience higher socioeconomic disadvantages that increase their risk of contracting COVID-19. People in higher socioeconomic status and affluent neighbourhoods have been reported to be less likely to acquire COVID-19 infection, whereas social and economic disadvantages have been associated with higher COVID-19 infections [99,100].

Evidence suggests that racial minorities in urban settings tend to live in more crowded conditions and are more likely to be employed in public-facing occupations (for example, services and transportation), making practising social distancing practically impossible [13,15,101]. For example, one study found that Blacks and Latinos working in essential services experienced higher COVID-19 mortality rates than Whites [102]. The authors observed that many Blacks held the top nine essential jobs that exposed them to the risk of catching COVID-19, increasing the potentials of infecting their families. Another study found that frontline jobs mainly occupied by Blacks and Hispanics were risk factors to COVID-19 [103]. Despite racial minorities being at increased risk of exposure to the virus, they experience increased barriers to testing, such as health care access, which contributes to delays in obtaining testing until they are in a more serious condition resulting in poor health outcomes [101].

Structural racism as a key determinant of population health can also explain the disproportionate burdens of COVID-19 found among racial minorities across the outcomes measured in the current study [104]. While there are differences between and within countries relating to the experience of structural racism and health inequities because of differences in policies and forces that facilitate these issues, these systems of oppression can lead to health inequities because of experienced disadvantages and lack of opportunities [105,106].

Blacks, Hispanics, and Other minority racial populations experience disproportionately higher rates of other underlying health conditions, making them more vulnerable to COVID-19. For example, a report found that neighbourhoods with higher poverty rates occupied by Blacks and Hispanics experienced disproportionate diabetes and hypertension comorbidities and higher rates of COVID-19 infections [103]. Other studies have shown that comorbidities such as obesity and cardiovascular diseases, where Blacks and Others are disproportionately overrepresented, were a risk factor for COVID-19 related mortality [6,107-109]. It may also be that older Blacks, Hispanics and Other racial minority groups have higher comorbidities than Whites, increasing their risks to COVID-19. Older age, regardless of race, has, however, been identified as a risk factor to COVID-19 [107,110,111].

Other factors, such as underlying health conditions and old age, could have contributed to the increased risk of COVID-19 in minority populations [42,60,112,113]. However, many underlying health conditions result from years of systemic inequities in social determinants of health, including poor housing, lack of employment, low income, racism, poor neighbourhood, and poor working and living conditions experienced by minority populations in western countries [112,114-117]. For example, residents in poor neighbourhoods with a poorly built environment to facilitate physical activities are less likely to participate in physical activities and are more likely to live sedentary lifestyles [118]. Sedentary lifestyles and lack of physical activities are risk factors for cardiovascular-related health conditions, including but not limited to hypertension, diabetes, and obesity [112]. In countries such as the United States, issues related to structural determinants of health are prevalent and disproportionately experienced by minority populations, which leads to generational inequities [99,119].

Given these findings, there is a need for a shift of focus from treating comorbidities from clinical perspectives only to addressing broader socioeconomic and structural determinants of health disadvantages experienced by most Blacks, Hispanics and Other minority groups in western countries [120]. Unprecedentedly, COVID-19 has exposed the social disadvantages that Blacks, Hispanics and Other minority populations continue to experience because of racism, discrimination, and systemic institutional policy of racial suppression in western countries [104]. Governments and policymakers have an opportunity to turn the course of health inequities in minority populations by investing in programs that facilitate equity in disadvantaged communities for improved health outcomes.

### Limitations and strengths

There are a few limitations to consider when interpreting the finding from this article. The included studies used different study designs and populations that could have influenced the selection of study participants and protocols, and thus, the findings. Some studies did not clearly describe whether race was self-reported and could have misclassified the race of some patients, which could have led to under-or over-reporting of COVID-19 outcomes. The included studies did not have data on many moderators, and we could not conduct a meta-analysis controlling for them. The different sources of data used by the different studies (for example, hospital data and sentinel data), differences in specificity and sensitivity of tests used could have contributed to the high heterogeneity observed among the studies, and the differences in the ascertainment of death outcomes are important limitations to note. Lastly, we did not search for all the grey literature, which could have inadvertently led to the omission of additional potential studies. It is, therefore, important to interpret our findings with these limitations in mind.

An important strength of this paper is its methodologically rigorous analysis that involved correcting for publication bias and synthesising the findings to highlight the extent of the disproportionate burden of COVID-19 in racial minority groups. This means that the findings cannot be attributed to publication bias which ensures confidence in the finding. Another strength of this review is its use of meta-regression analysis controlling for the different parameters, and this was vital to compare the results by sub-groups. Our findings highlight the disproportionate burdens of COVID-19 outcomes among the selected race groups.

## CONCLUSIONS

The burden of COVID-19 in terms of prevalence, hospitalisation, and mortality rate was disproportionately higher among Blacks and Hispanics compared with Whites. These findings point to the systemic disadvantages experienced by racial minority populations and highlight the need to address inequities in these communities by developing programs that improve overall health outcomes. Further work and more well-designed longitudinal studies are needed to expand the knowledge on racial differences in COVID-19 outcomes and to identify the social determinants of health shaping the disparities in the outcome of COVID-19 among racial minority populations.

## Additional material

Online Supplementary Document
