# Supplementary material for: Racial disparities in COVID-19 pandemic cases, hospitalisations, and deaths: A systematic review and meta-analysis
Source: J Glob Health. 2021 Jun 26;11:05015. doi: 10.7189/jogh.11.05015 (PMC8248751; doi:10.7189/jogh.11.05015)
Supplement: Online Supplementary Document [file jogh-11-05015-s001.zip › Supplementary materials/Online Supplementary Documents.docx]

# Supplementary materials:

**Table S1:** Included studies reporting cases, hospitalisations, and deaths from COVID-19 by race.

**Figure S1**: Standardised Prevalence Ratio (SPR) Funnel Plots by race. Panel A shows SPR funnel plot for Blacks. Panel B shows SPR funnel plot for Hispanics. Panel C shows SPR funnel plot for Other race groups. Panel D shows SPR funnel plot for Whites.

**Figure S2**: Standardised hospitalisation Ratio (SHR) Funnel Plots by race. Panel A shows SHR funnel plot for Blacks. Panel B shows SHR funnel plot for Hispanics. Panel C shows SHR funnel plot for Other race groups. Panel D shows SHR funnel plot for Whites.

**Figure S3**: Standardised Mortality Ratio (SMR) Funnel Plots. Panel A shows SMR funnel plot for Blacks. Panel B shows SMR funnel plot for Hispanics. Panel C shows SMR funnel plot for Other race groups. Panel D shows SMR funnel plot for Whites.

**Appendix S1:** Trim and Fill for Standardised Prevalence Ratio (SPR) by race .

**Appendix S2:** Trim and Fill for Standardised Hospitalisation Ratio (SHR) by race.

**Appendix S3:** Trim and Fill for Standardised Mortality Ratio (SMR) by race.
