# Supplementary figures and images for: Racial disparities in COVID-19 pandemic cases, hospitalisations, and deaths: A systematic review and meta-analysis
Source: J Glob Health. 2021 Jun 26;11:05015. doi: 10.7189/jogh.11.05015 (PMC8248751; doi:10.7189/jogh.11.05015)

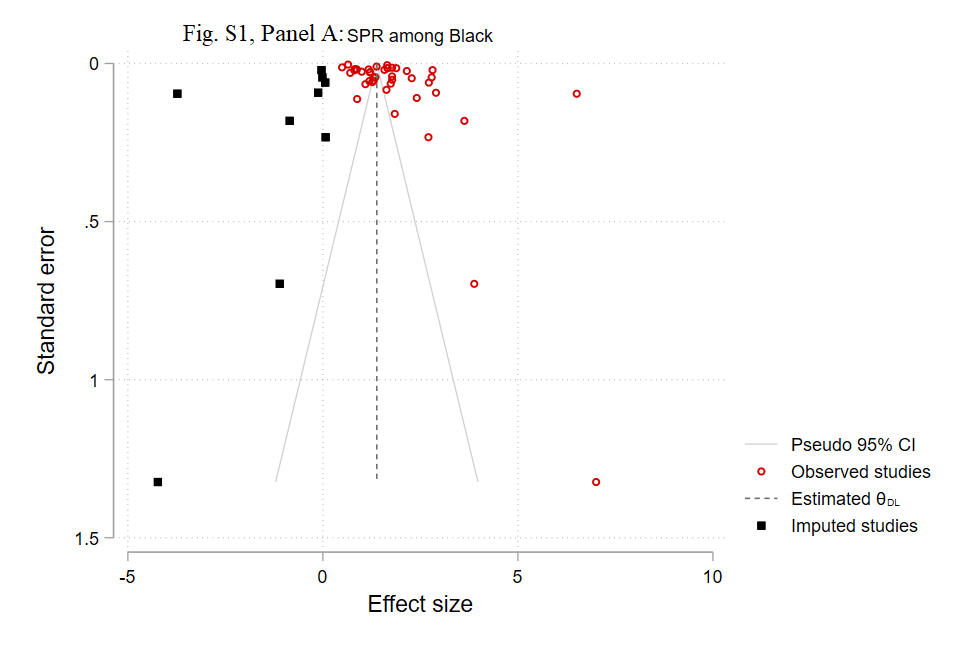

Supplement: Online Supplementary Document [file jogh-11-05015-s001.zip › Supplementary materials/Figure S1/Fig S1 Panel A_SPR Funnel Plot for Black.tif]

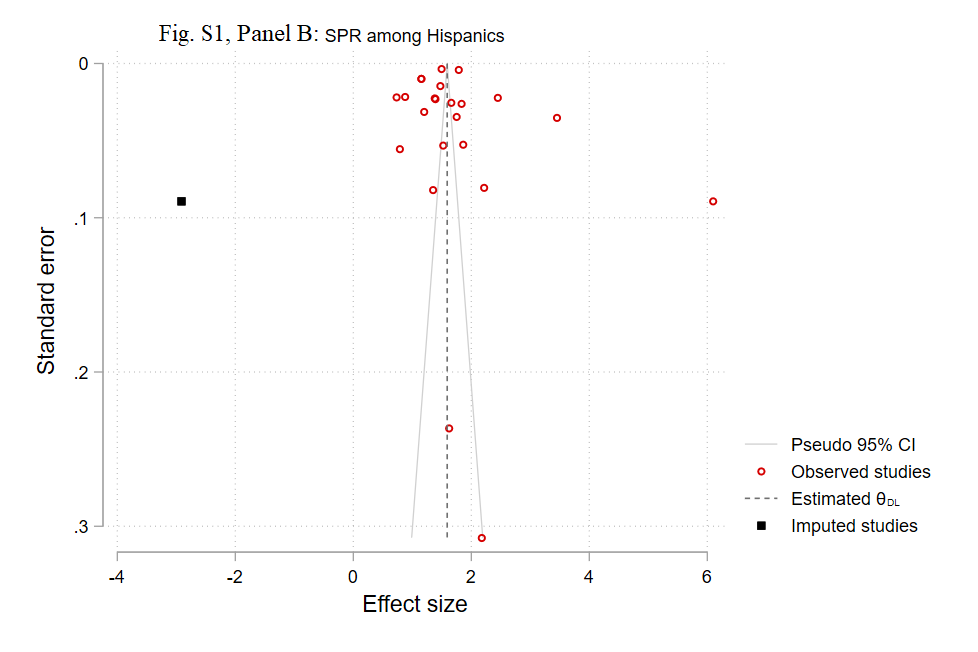

Supplement: Online Supplementary Document [file jogh-11-05015-s001.zip › Supplementary materials/Figure S1/Fig S1 Panel B_SPR Funnel Plot for Hispanics.tif]

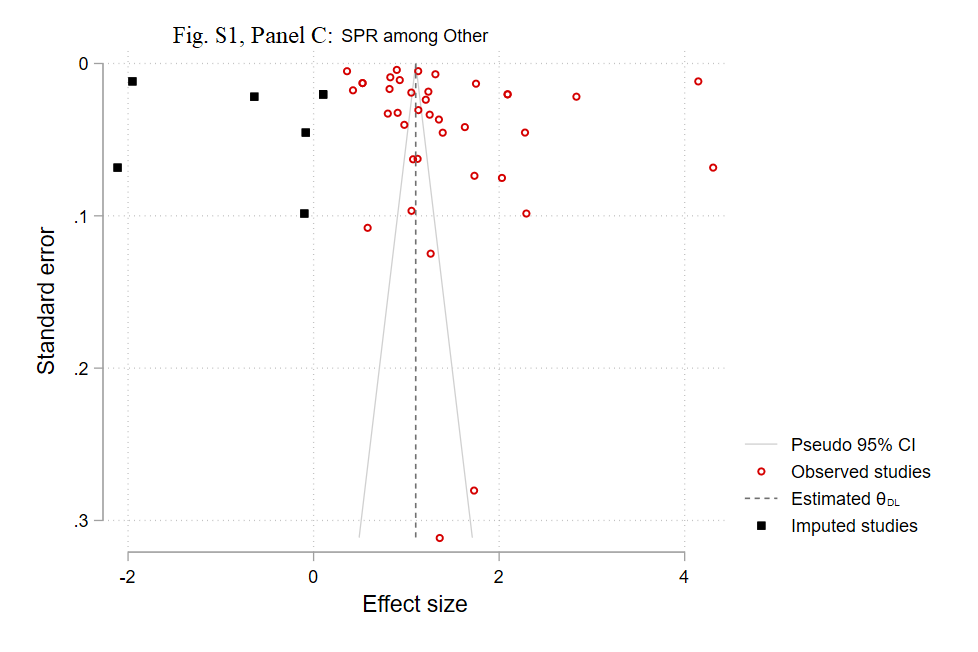

Supplement: Online Supplementary Document [file jogh-11-05015-s001.zip › Supplementary materials/Figure S1/Fig S1 Panel C_SPR Funnel Plot for Other.tif]

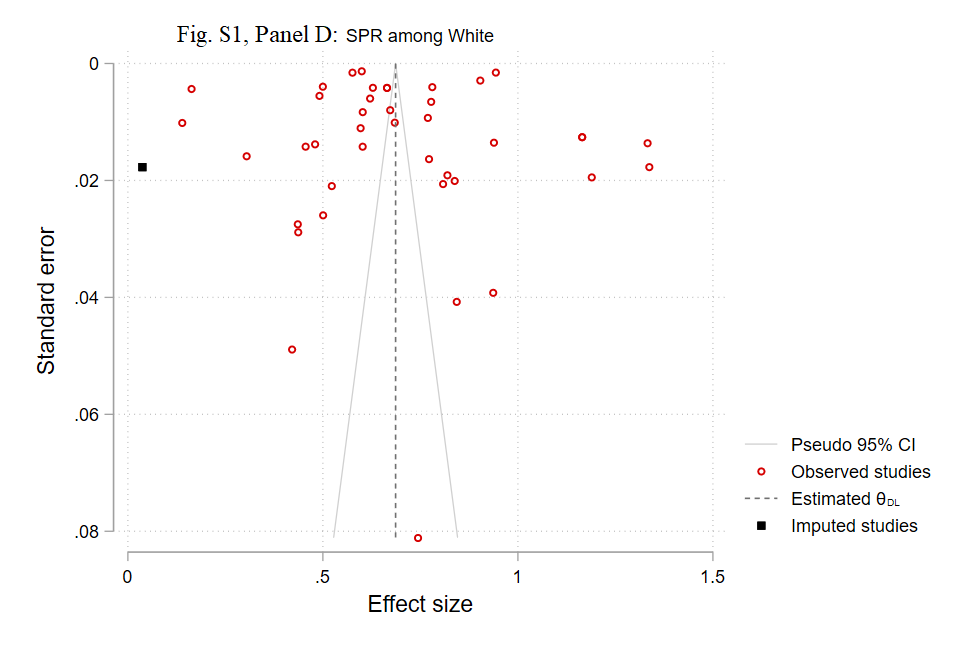

Supplement: Online Supplementary Document [file jogh-11-05015-s001.zip › Supplementary materials/Figure S1/Fig S1 Panel D_SPR Funnel Plot for White.tif]

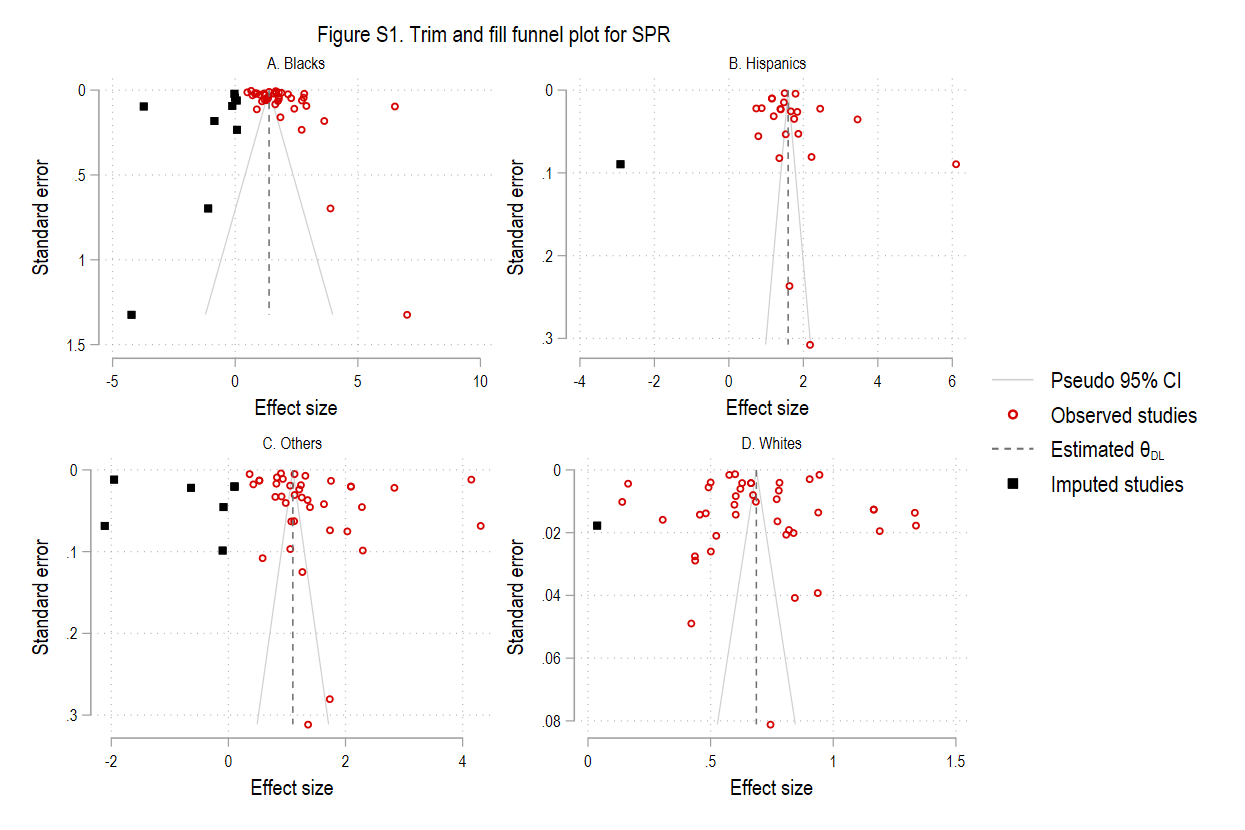

Supplement: Online Supplementary Document [file jogh-11-05015-s001.zip › Supplementary materials/Figure S1/Trim_SPR.tif]

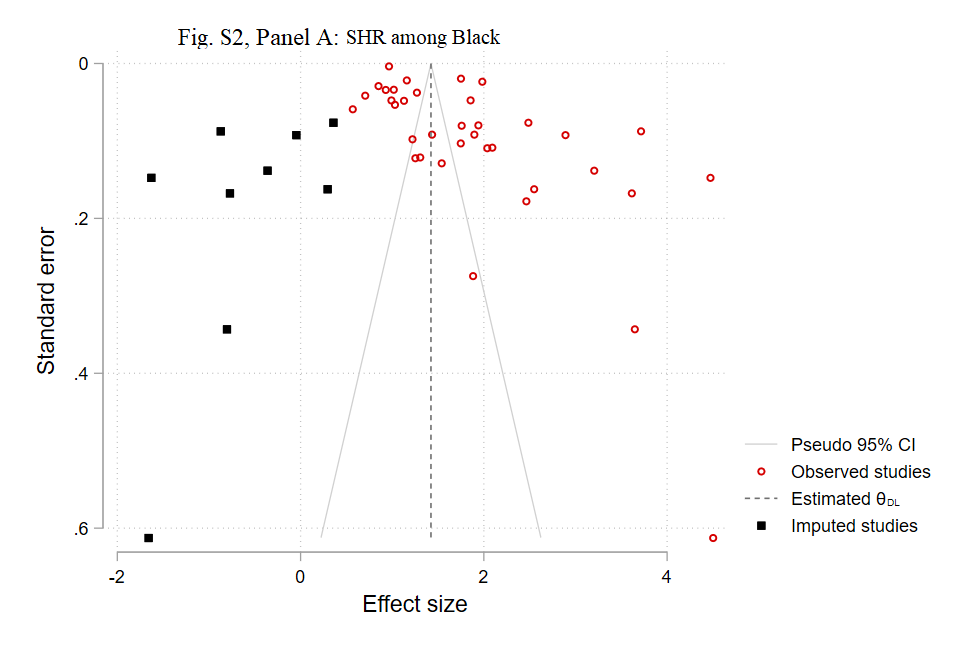

Supplement: Online Supplementary Document [file jogh-11-05015-s001.zip › Supplementary materials/Figure S2/Fig S2 Panel A_SHR Funnel Plot for Black.tif]

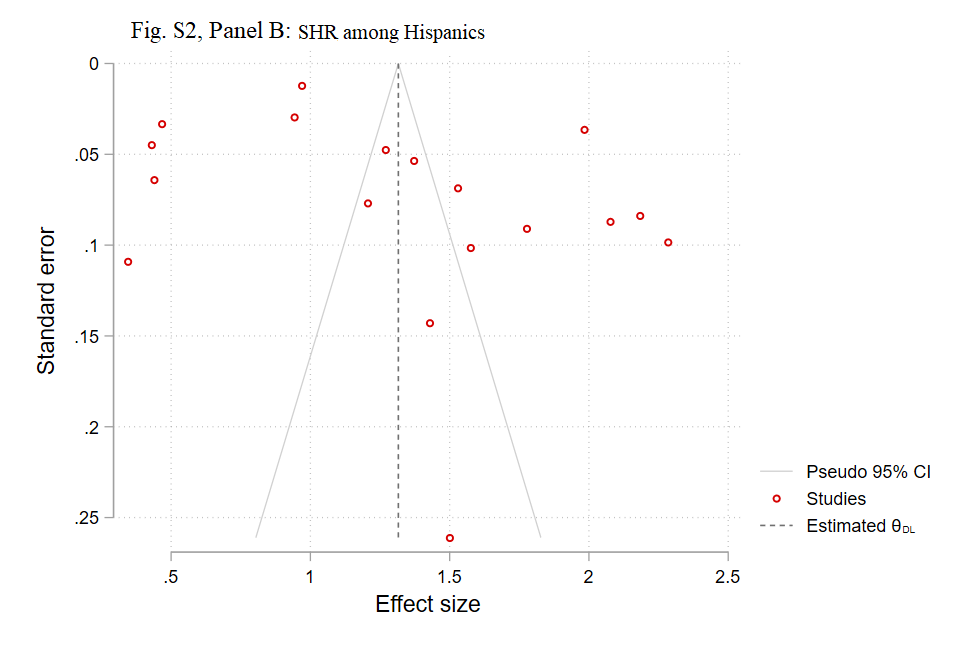

Supplement: Online Supplementary Document [file jogh-11-05015-s001.zip › Supplementary materials/Figure S2/Fig S2 Panel B_SHR Funnel Plot for Hispanics.tif]

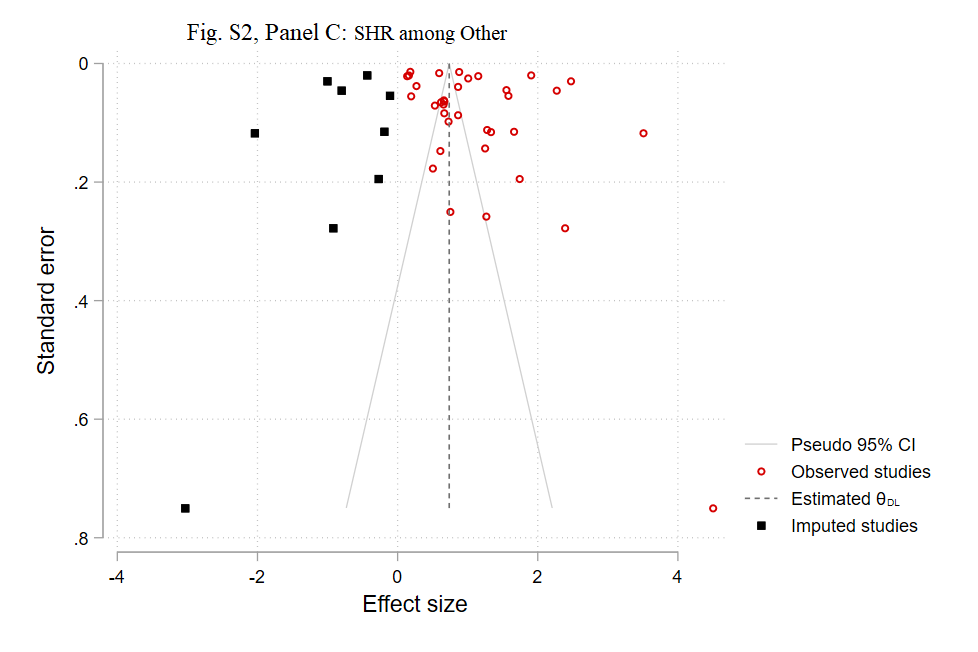

Supplement: Online Supplementary Document [file jogh-11-05015-s001.zip › Supplementary materials/Figure S2/Fig S2 Panel C_SHR Funnel Plot for Other.tif]

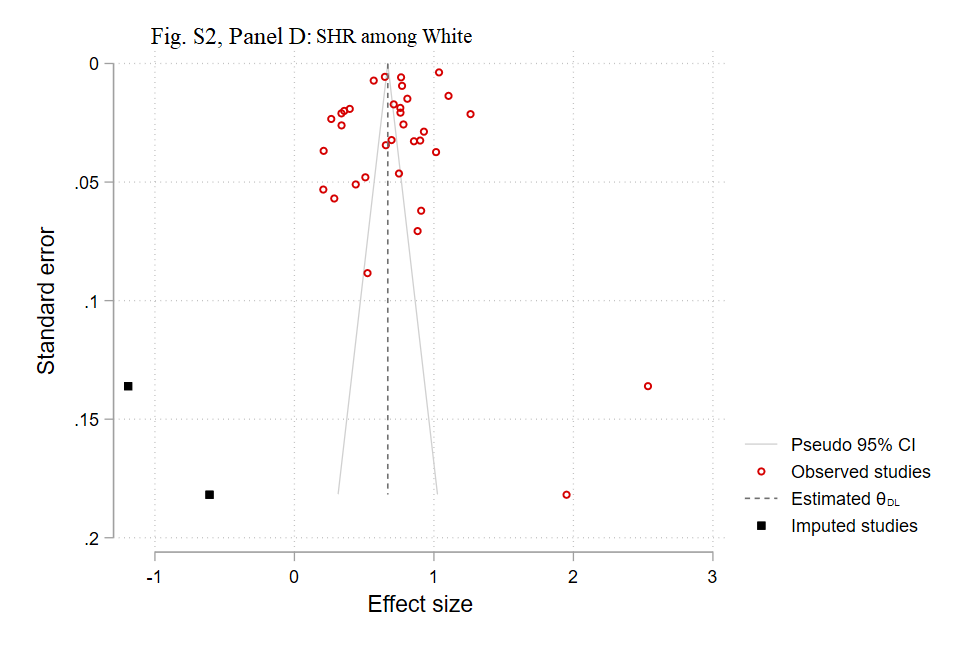

Supplement: Online Supplementary Document [file jogh-11-05015-s001.zip › Supplementary materials/Figure S2/Fig S2 Panel D_SHR Funnel Plot for White.tif]

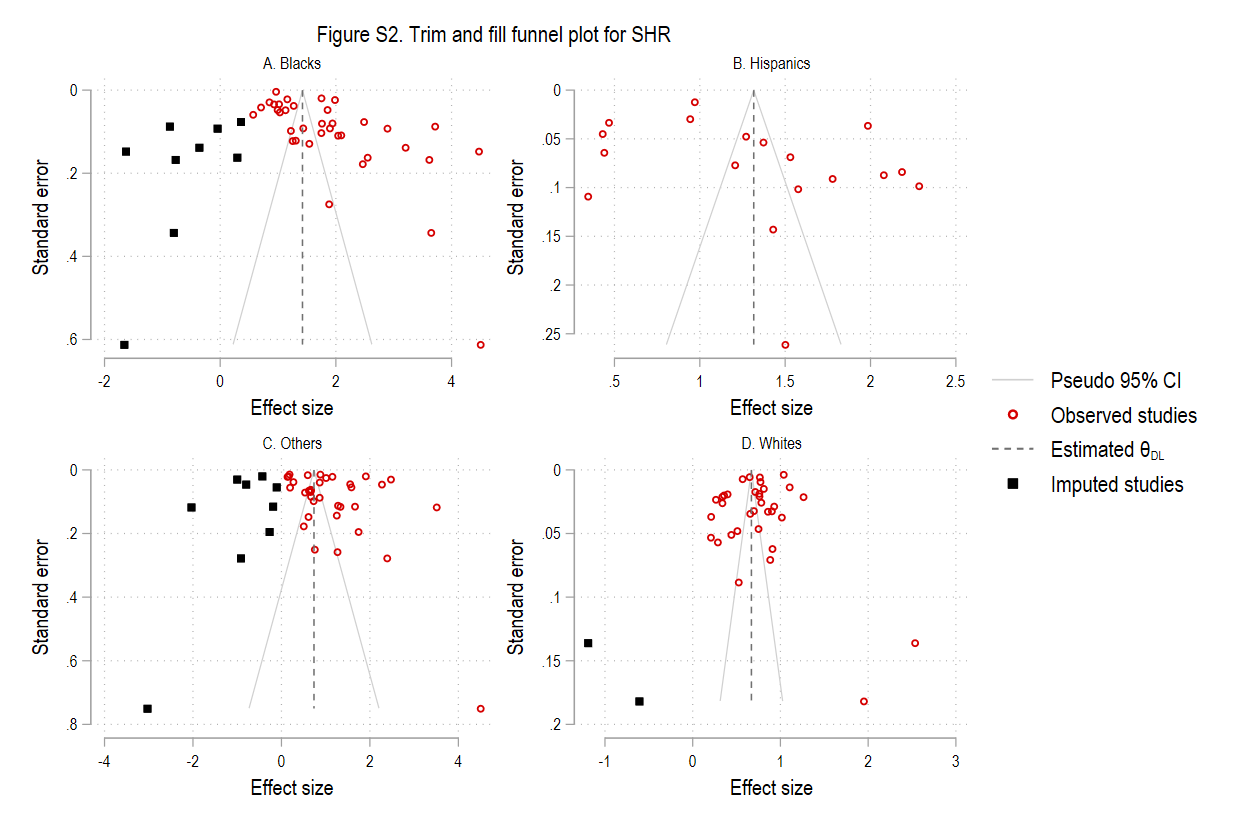

Supplement: Online Supplementary Document [file jogh-11-05015-s001.zip › Supplementary materials/Figure S2/Trim_SHR.tif]

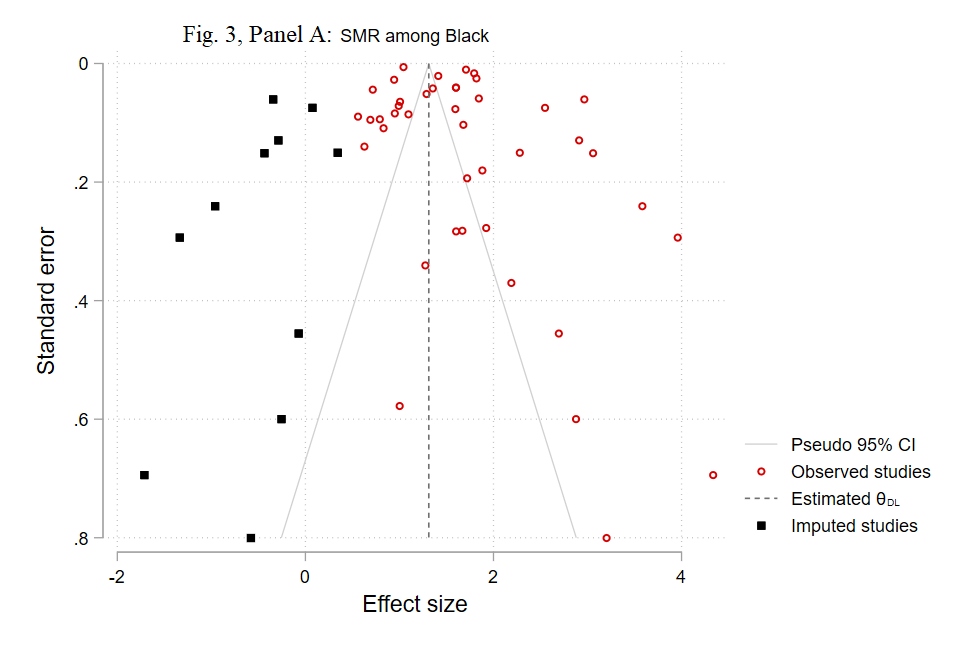

Supplement: Online Supplementary Document [file jogh-11-05015-s001.zip › Supplementary materials/Figure S3/Fig S3 Panel A_SMR Funnel Plot for Black.tif]

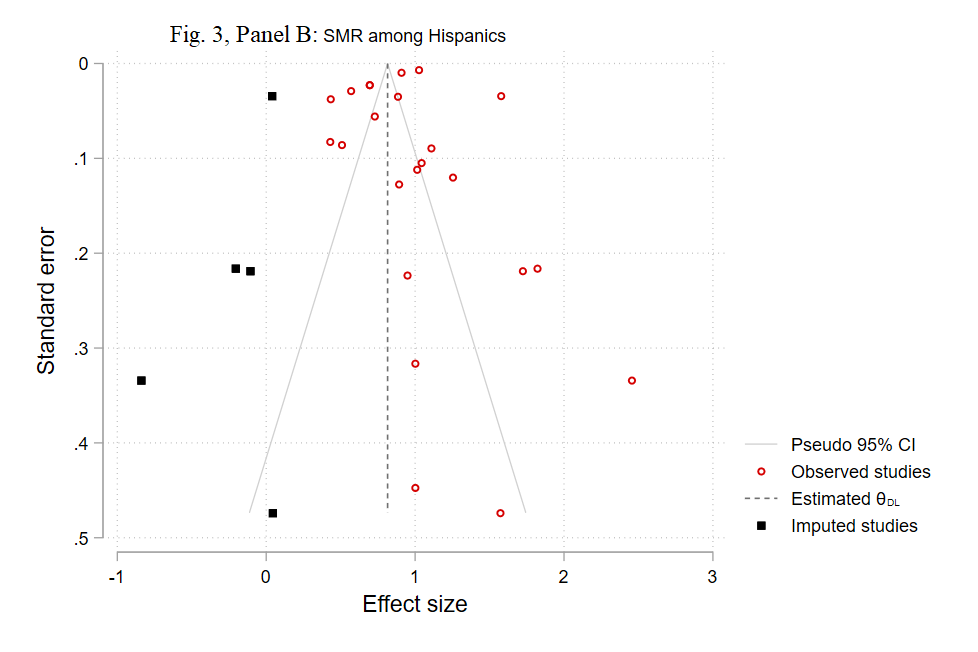

Supplement: Online Supplementary Document [file jogh-11-05015-s001.zip › Supplementary materials/Figure S3/Fig S3 Panel B_SMR Funnel Plot for Hispanics.tif]

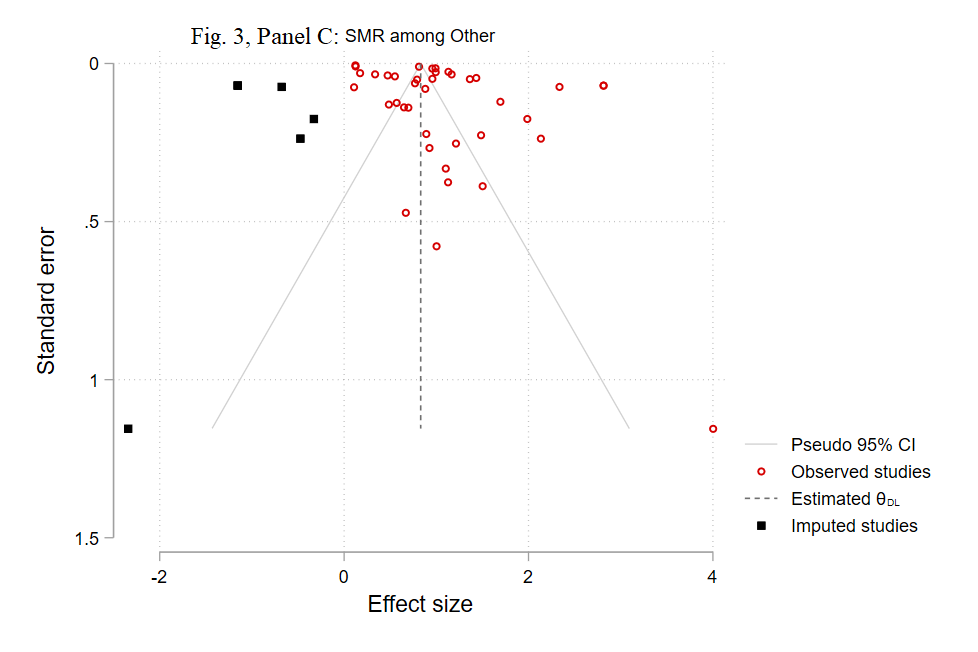

Supplement: Online Supplementary Document [file jogh-11-05015-s001.zip › Supplementary materials/Figure S3/Fig S3 Panel C_SMR Funnel Plot for Other.tif]

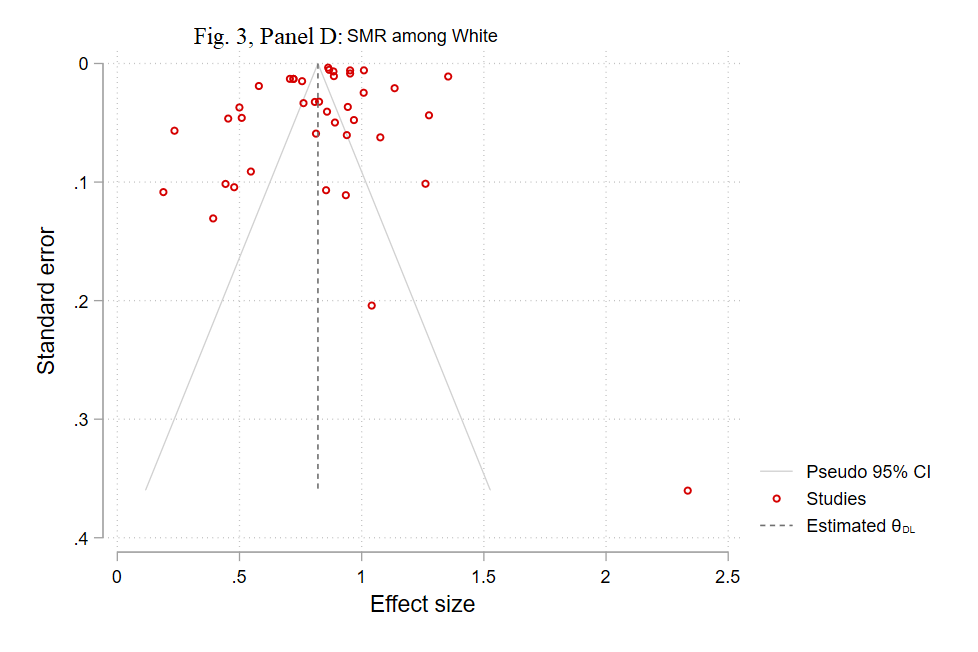

Supplement: Online Supplementary Document [file jogh-11-05015-s001.zip › Supplementary materials/Figure S3/Fig S3 Panel D_SMR Funnel Plot for White.tif]

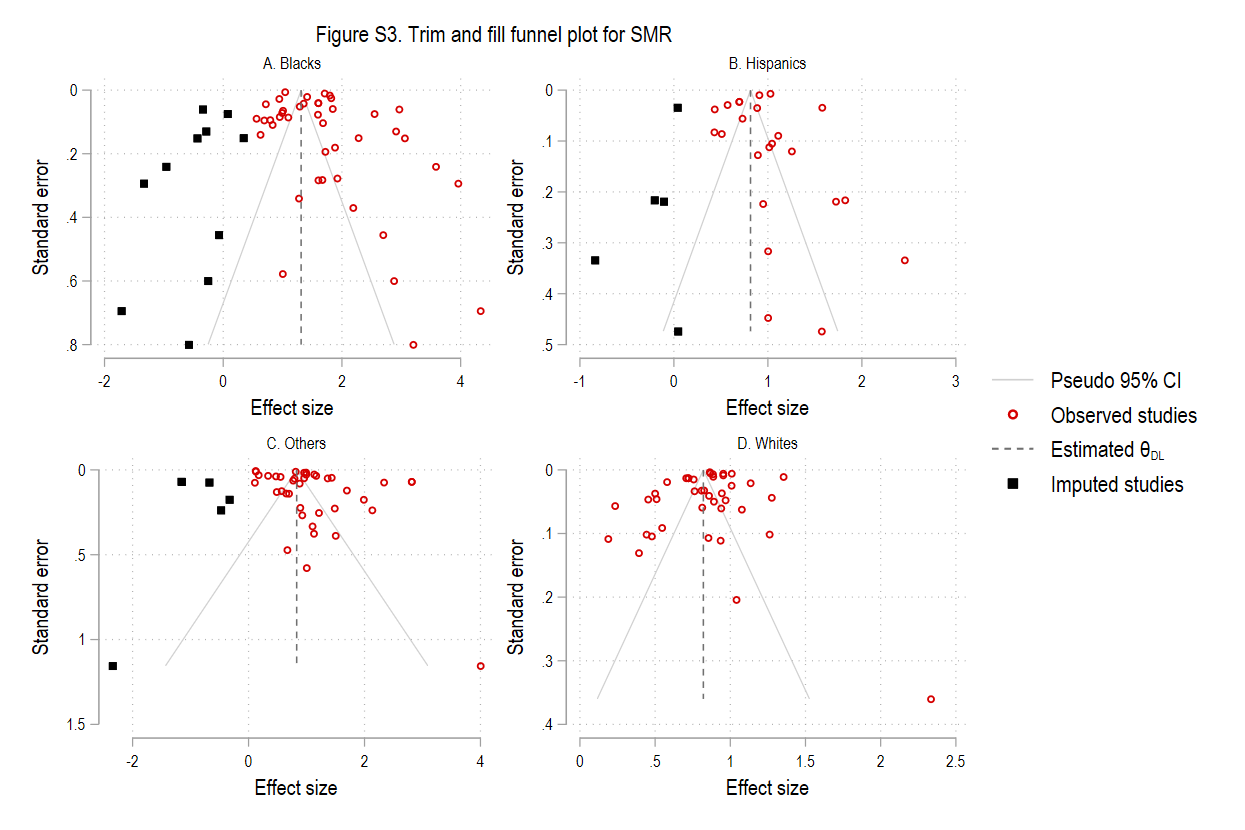

Supplement: Online Supplementary Document [file jogh-11-05015-s001.zip › Supplementary materials/Figure S3/Trim_SMR.tif]
